# Transcriptomic analysis of the oleaginous yeast *Lipomyces starkeyi* during lipid accumulation on enzymatically treated corn stover hydrolysate

**DOI:** 10.1186/s13068-019-1510-z

**Published:** 2019-06-26

**Authors:** Kyle R. Pomraning, James R. Collett, Joonhoon Kim, Ellen A. Panisko, David E. Culley, Ziyu Dai, Shuang Deng, Beth A. Hofstad, Mark G. Butcher, Jon K. Magnuson

**Affiliations:** 1 Pacific Northwest National Laboratory, Richland, WA USA; 2 Joint BioEnergy Institute, Emeryville, CA USA

**Keywords:** *Lipomyces starkeyi*, Oleaginous, Yeast, Bioreactor, Biofuel, Corn stover, PCS, Inhibitors, Transcriptome

## Abstract

**Background:**

Efficient and economically viable production of biofuels from lignocellulosic biomass is dependent on mechanical and chemical pretreatment and enzymatic hydrolysis of plant material. These processing steps yield simple sugars as well as plant-derived and process-added organic acids, sugar-derived dehydration products, aldehydes, phenolics and other compounds that inhibit the growth of many microorganisms. *Lipomyces starkeyi* is an oleaginous yeast capable of robust growth on a variety of sugars and lipid accumulation on pretreated lignocellulosic substrates making it attractive as an industrial producer of biofuels. Here, we examined gene expression during batch growth and lipid accumulation in a 20-L bioreactor with either a blend of pure glucose and xylose or pretreated corn stover (PCS) that had been enzymatically hydrolyzed as the carbon sources.

**Results:**

We monitored sugar and ammonium utilization as well as biomass accumulation and found that growth of *L. starkeyi* is inhibited with PCS hydrolysate as the carbon source. Both acetic acid and furfural are present at concentrations toxic to *L. starkeyi* in PCS hydrolysate. We quantified gene expression at seven time-points for each carbon source during batch growth and found that gene expression is similar at physiologically equivalent points. Analysis of promoter regions revealed that gene expression during the transition to lipid accumulation is regulated by carbon and nitrogen catabolite repression, regardless of carbon source and is associated with decreased expression of the translation machinery and suppression of the cell cycle. We identified 73 differentially expressed genes during growth phase in the bioreactor that may be involved in detoxification of corn stover hydrolysate.

**Conclusions:**

Growth of *L. starkeyi* is inhibited by compounds present in PCS hydrolysate. Here, we monitored key metabolites to establish physiologically equivalent comparisons during a batch bioreactor run comparing PCS hydrolysate and purified sugars. *L. starkeyi*’s response to PCS hydrolysate is primarily at the beginning of the run during growth phase when inhibitory compounds are presumably at their highest concentration and inducing the general detoxification response by *L. starkeyi*. Differentially expressed genes identified herein during growth phase will aid in the improvement of industrial strains capable of robust growth on substrates containing various growth inhibitory compounds.

**Electronic supplementary material:**

The online version of this article (10.1186/s13068-019-1510-z) contains supplementary material, which is available to authorized users.

## Background

The finite and decreasing supply of petroleum available for human utilization has prompted the development of sustainable methods to produce hydrocarbons. Processes to convert lignocellulosic biomass into simple sugars that can be fed to robust microorganisms to produce biofuels are an attractive route to achieve this. Oleaginous fungi capable of producing high quantities of lipids can be used to convert lignocellulosic biomass into biodiesel or hydrocarbon fuel-blending agents utilizable within the petrochemical industry infrastructure. Oleaginous yeasts in particular from the genera *Lipomyces*, *Rhodosporidium*, and *Yarrowia* have proven to be robust lipid accumulators that can be readily engineered for improvement of titer, rate, and yield as well as production of synthetic lipid-derived compounds [1–3].

Rapid conversion of lignocellulosic biomass into biofuels requires that the plant material be physically, chemically, and/or enzymatically broken down to release simple sugars. A wide variety of methods are available to achieve this and result in a spectrum of chemicals derived from the plant biomass, dependent on the pretreatment conditions, along with the simple sugars [4–6]. Dilute acid pretreatment followed by enzymatic digestion was one of the earliest successful strategies for production of simple sugars from lignocellulosic biomass [7, 8] and remains relevant for commercial production of biofuels [9–11]. The process is rapid and efficient but yields sugars containing growth and conversion inhibitors such as acetic acid, cinnamic acids, furfural, hydroxymethylfurfural, and phenolic compounds [6, 12–14]. Recent efforts have been made to engineer and evolve bioconversion organisms such as *Saccharomyces cerevisiae* [10, 15, 16], *Bacillus coagulans* [11], *Zymomonas mobilis* [17], *Acinetobacter baylyi* [18], *Caldicellulosiruptor bescii* [19], and *Rhodococcus opacus* [20] among others for improved growth in the presence of these inhibitory compounds. However, a number of fungi capable of robust growth in the presence of pretreatment derived inhibitors have been identified [21, 22]. *Lipomyces starkeyi* stands out as an oleaginous yeast [23] capable of growth on a wide range of carbon and nitrogen sources and pretreated feedstocks [24–27] in addition to being genetically tractable [28–31].

Here, we applied a systematic approach to characterize the response of *L. starkeyi* to the plethora of compounds that are present in pretreated corn stover enzymatic hydrolysate (PCS) in addition to the primary carbohydrates glucose and xylose. *L. starkeyi* was grown in a bioreactor in batch mode for production of lipids with all variables held constant with the exception of the carbon source. Online monitoring of biologically relevant parameters and nutrient concentrations was used to sample the bioreactor at physiologically equivalent time-points (e.g., prior to and after depletion of specific nutrients during batch cultivation) and assay gene expression by RNA sequencing. This is the first thorough analysis of the response to PCS performed in oleaginous yeast. We found that comparing growth on PCS and clean sugars at equivalent physiological states during a batch lipid accumulation run, rather than at equivalent time-points, greatly expanded our understanding of the response to PCS in *L. starkeyi* by eliminating biases attributable to differences in growth phase and concentration of extracellular carbon sources and other nutrients.

## Materials and methods

### Chemicals, growth media and yeast strains

All chemicals and reagents were purchased from Sigma-Aldrich (St. Louis, MO) unless otherwise noted. *L. starkeyi* strain NRRL Y-11558 was obtained from American Type Tissue Culture (ATCC^®^64135; Manassas, VA) and was used for all experiments in this study. *L. starkeyi* was maintained on YPD plates (1% yeast extract, 1% peptone, 2% glucose, 2% agar) at 28 °C. Frozen stocks were maintained at − 80 °C in 15% glycerol. For bioreactor experiments, *L. starkeyi* was pre-grown from glycerol stocks in 50-mL YPD broth in 250-mL shake flasks at 30 °C and 200 rpm. Saccharified dilute acid pretreated corn stover (PCS; lot CH131104) was provided as a slurry with 60.2% moisture by Dan Schell of the National Renewable Energy Laboratory (NREL; Golden, CO), along with the compositional analysis of the PCS shown in Table 1.

**Table 1:** Composition of pretreated corn stover used in this study

Solids composition (% w/w dry basis)									
Ash	Protein	Lignin	Glucan	Xylan	Galactan	Arabinan	Mannan	Acetate	
2.4	1.8	27.9	60.7	3.8	ND	0.7	ND	0.3	

Composition of liquor fraction (g/L)									
Lignin	Cellobiose	Glucose	Xylose	Galactose	Arabinose	Fructose	Acetate	HMF	Furfural
9.94	2.66	21.38	157.74	9.81	19.48	ND	13.2	0.5	4.08

PCS medium used in the experiments was prepared by suspending 3 kg of the PCS slurry into ~ 20 L of distilled water. The diluted slurry was passed through a coarse screen mesh to remove rocks and other large debris, and then hydrolyzed with CTEC2 and HTEC2 enzymes (Novozymes; Franklinton, NC) at 50 °C for 96 h. Prior to enzymatic hydrolysis, the pH of the diluted PCS had been raised to 5.0 via the addition of 5-M KOH. The hydrolyzed PCS was harvested from the bioreactor and then passed through a CEPA continuous centrifuge spinning at 20,000 RPM to remove fine-suspended lignin and other particles. Enzymatic hydrolysis of PCS yielded 54.0 g/L glucose, 3.3 g/L acetic acid, 0.092 g/L hydroxymethylfurfural, and 1.25 g/L furfural. A portion of the clarified supernatant was diluted with distilled water to prepare MMPCS media with a targeted glucose concentration of 18.5 g/L with 100% of the sugars in the medium coming from the hydrolyzed PCS. The MMPCS medium was supplemented with 1.5 g/L KH_2_PO_4_, 0.62 g/L NH_4_Cl, 0.5 g/L KCl, 0.5 g/L MgSO_4_·7H_2_O, and 1 mg/L each of biotin, pyridoxine, thiamine, riboflavin, para-aminobenzoic acid, and nicotinic acids, along with 1 mL/L of a 1000× trace elements solution containing 2.25 g/L ZnSO_4_·7H_2_O, 11 g/L H_3_BO_3_, 5 g/L MnCl_2_·4H_2_O, 5 g/L FeSO_4_·7H_2_O, 1.7 g/L CoCl_2_·6H_2_O, 1.6 g/L CuSO_4_·5H_2_0, 0.085 g/L Na_2_MoO_4_·2H_2_O, and 5 g/L Na_4_EDTA. The initial concentration of potentially inhibitory compounds in this medium after dilution is 1.14 g/L acetic acid, 0.032 g/L hydroxymethylfurfural, and 0.43 g/L furfural. A synthetic medium identified as MMGX was prepared with identical sugar concentrations and nutrient supplements to the MMPCS medium (18.5 g/L glucose, 8.7 g/L xylose, 1.5 g/L KH_2_PO_4_, 0.62 g/L NH_4_Cl, 0.5 g/L KCl, 0.5 g/L MgSO_4_·7H_2_O, and 1 mg/L each of biotin, pyridoxine, thiamine, riboflavin, para-aminobenzoic acid, and nicotinic acids, along with 1 mL/L of a 1000× trace elements solution containing 2.25 g/L ZnSO_4_·7H_2_O, 11 g/L H_3_BO_3_, 5 g/L MnCl_2_·4H_2_O, 5 g/L FeSO_4_·7H_2_O, 1.7 g/L CoCl_2_·6H_2_O, 1.6 g/L CuSO_4_·5H_2_0, 0.085 g/L Na_2_MoO_4_·2H_2_O, and 5 g/L Na_4_EDTA) but with the sugars provided by the addition of purified glucose and xylose powders instead of the PCS slurry.

### Bioreactor cultivation of *L. starkeyi*

Submerged stirred tank cultivations of *L. starkeyi* cells were performed using either MMPCS or MMGX growth medium in 20 L of media within a 30-L Sartorius Biostat-C bioreactor, under the following identical conditions: the bioreactor was charged with medium and sterilized at 121 °C for 30 min. The medium pH was adjusted to 6.0 and then maintained at a minimum of 5.5 via the automated addition of 5-M KOH. The temperature was maintained at 30 °C, the stirring speed was set at a constant rate of 600 RPM, and the dissolved oxygen concentration was maintained at 50% via the automated control of sparging air flow, which varied between 1 and 30 LPM during the course of the experiments. The bioreactor was inoculated with washed *L. starkeyi* cells (that had been grown in shake flasks as described above) to achieve a concentration of 10^6^ cells/mL in the bioreactor vessel. Samples of 50–100 mL in volume were taken from the bioreactor broth at regular intervals during the experiments. 1 mL of the broth was immediately dispensed into each of two cryotubes and immediate-frozen in liquid nitrogen for later transcriptomic analysis. Another 4 mL of the broth was passed through a 0.2 μm filter and then frozen at − 20 °C for later analysis of sugar, nutrient, and metabolite concentrations. The remaining broth was centrifuged at 1825×*g* to pellet the yeast cells, which were then washed in distilled water and centrifuged again. The supernatant was discarded and the pelleted cells were then stored at − 80 °C. The pelleted cells were later lyophilized and their dry cell mass was measured gravimetrically. Aliquots of the dry cell mass were taken for measurement of intracellular lipid concentration, as described below.

### Bioreactor broth analysis

Changes in the concentrations of sugar substrates during the course of the bioreactor and shake flask cultivations were determined via high-pressure liquid chromatography (HPLC) analysis. Samples of the centrifuged culture supernatant were thawed to room temperature, vigorously mixed, and then serially diluted. From each dilution, a 10-µL injection of sample into an Agilent 1200 HPLC system equipped with a BEH Amide column (Waters XBridge, 130 Å, 3.5 μm, 4.6 mm × 250 mm) and a guard column (Waters XBridge, 130 Å, 3.5 μm, 4.6 mm × 20 mm) that were maintained at 30 °C. An isocratic solution of 40% acetone, 40% acetonitrile, 0.1% ammonium hydroxide at 0.6 mL/min was used to elute samples from the column into an evaporative light scattering detector (ELSD, Alltech ELSD 2000). The ELSD settings were as follows: tube temperature, 63 °C; nebulizer gas flowrate, 1.5 mL/min; and gain, 8. The ELSD signal was converted from analog to digital using a dual-channel interface (Agilent 35900E) for integration with the HPLC software. Calibration was performed on sugars dissolved in water using the peak area of the HPLC chromatogram. For each sugar, the data were analyzed at five different weight percent levels with quadruplicate measurements for each level in a blocked randomized design. Triplicate measurements were performed on each sample in a blocked randomized design with triplicate measurements of check standards at three different weight percent levels (low, medium and high) to validate instrument performance over the course of the analyses. Preliminary measurements of sugar substrate concentrations and measurements of bioreactor broth ammonia concentrations were made using a YSI 2950 Biochemistry Analyzer (Yellow Springs Instruments).

### Intracellular lipid quantification

From each previously frozen culture sample, 100 mg of dried cells was taken and then mixed with an internal standard consisting of 40 mg of a tridecanoic acid (C13:0) in 0.5 mL of methanol, along with 0.35 mL of 10-N KOH and another 2.65 mL of methanol. This mixture was incubated in a capped 50-mL Pyrex glass tube in a water bath shaker at 55 °C for 1.5 h. After incubation, the tube was cooled under cold tap water. 0.29 mL of 24 N H_2_SO_4_ was then added to the tube, which was inverted for mixing multiple times until a white K_2_SO_4_ precipitate began to form. To complete FAME synthesis, the tube was again incubated at 55 °C for 1.5 h while shaking. The capped tube was again cooled under tap water. The FAMEs were extracted from the cell mixture via the addition of 10 mL of hexane to the tube, which was agitated on a Vortex mixer for 5 min, and then centrifuged at 2750×*g* for 5 min. Aliquots of the supernatant were then submitted in triplicate for gas chromatography (GC) analysis.

### Transcriptomic sample preparation and analysis

RNA was extracted from samples using a Maxwell 16 LEV Plant RNA kit (Promega, Madison, WI) and sequenced on an Illumina platform. Sequences were mapped to the *L. starkeyi* NRRL-11557 genome [24] to predict transcription start sites and quantify expression. Clustering analyses were performed in R and expression level of genes quantified with featureCounts [32] and converted into reads per kilobase million (RPKM) (Additional file 1). Samples belonging to growth, transition, or stationary phase for each bioreactor run were treated as replicates to identify genes significantly differentially expressed in MMPCS versus MMGX using DESeq2 (adjusted *p* value < 0.01 and log_2_ fold-change > 1) [33]. Gene ontology analysis was performed with FunRich [34]. Metabolic modeling and biochemical pathway visualization utilized Cytoscape3 [35]. Promoter enrichment analysis was performed using DREME [36], Tomtom [37].

## Results

### Toxicity of inhibitory compounds present in pretreated corn stover

**Fig. 1:**
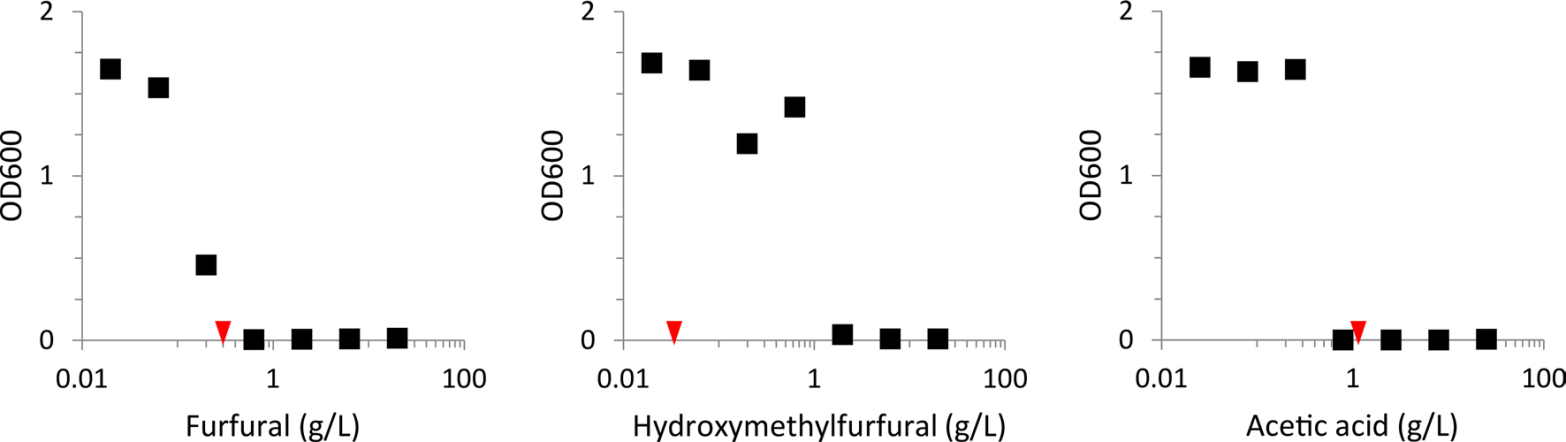
Growth of *L. starkeyi* in the presence of inhibitory compounds present in PCS. *L. starkeyi* was inoculated to an optical density of 600 nm (OD600) of 0.01 in liquid MMGX medium with addition of furfural, hydroxymethylfurfural, or acetic acid. OD600 was measured after 3 days of growth in a shaking incubator at 30 °C and 200 rpm. No additional growth was observed from low OD600 flasks when reassessed at day 7. Red arrows indicate the concentration of each inhibitor in the MMPCS medium after enzymatic hydrolysis. Results are representative of three replicates

Production of biofuels such as green diesel from alternative and renewable resources is desirable to reduce the carbon footprint of liquid hydrocarbon transportation fuels. Here, we investigated the use of PCS as a source of carbon (primarily in the form of glucose and xylose) for the production of lipid-derived biofuels. Economically viable processes to produce carbon accessible in the form of sugar monomers from cellulosic biomass typically result in extraction or generation of inhibitory compounds. In the case of PCS, these include small organic acids, lignin-derived compounds, and sugar dehydration products, such as, furfural, and hydroxymethylfurfural. We assessed the presence and concentration of inhibitory compounds in raw PCS lot CH131104 (Table 1), and examined the effect of these compounds on growth of *L. starkeyi* by spiking them individually into MMGX medium along with a small initial population of cells and assessing biomass production in shake flasks after 3 and 7 days. We found that *L. starkeyi* was able to grow in up to 0.2 g/L furfural, 0.63 g/L hydroxymethylfurfural, and 0.25 g/L acetic acid (Fig. 1). While furfural, hydroxymethylfurfural, and acetic acid are all toxic to *L. starkeyi*, only furfural and acetic acid are present at potentially toxic concentrations in PCS.

### Identification of the response to PCS using a batch lipid accumulation strategy

**Fig. 2:**
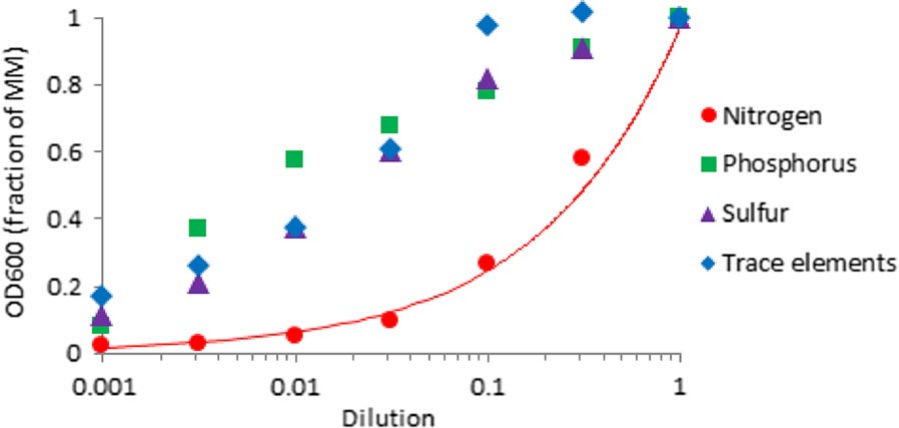
Growth-limiting nutrients in MMGX medium. *L. starkeyi* was inoculated to an OD600 of 0.001 in MMGX modified to reduce the concentration of specific nutrients and grown for a week in shake flasks at 30 °C and 200 rpm. Biomass production is most sensitive to the concentration of nitrogen in MMGX medium

**Fig. 3:**
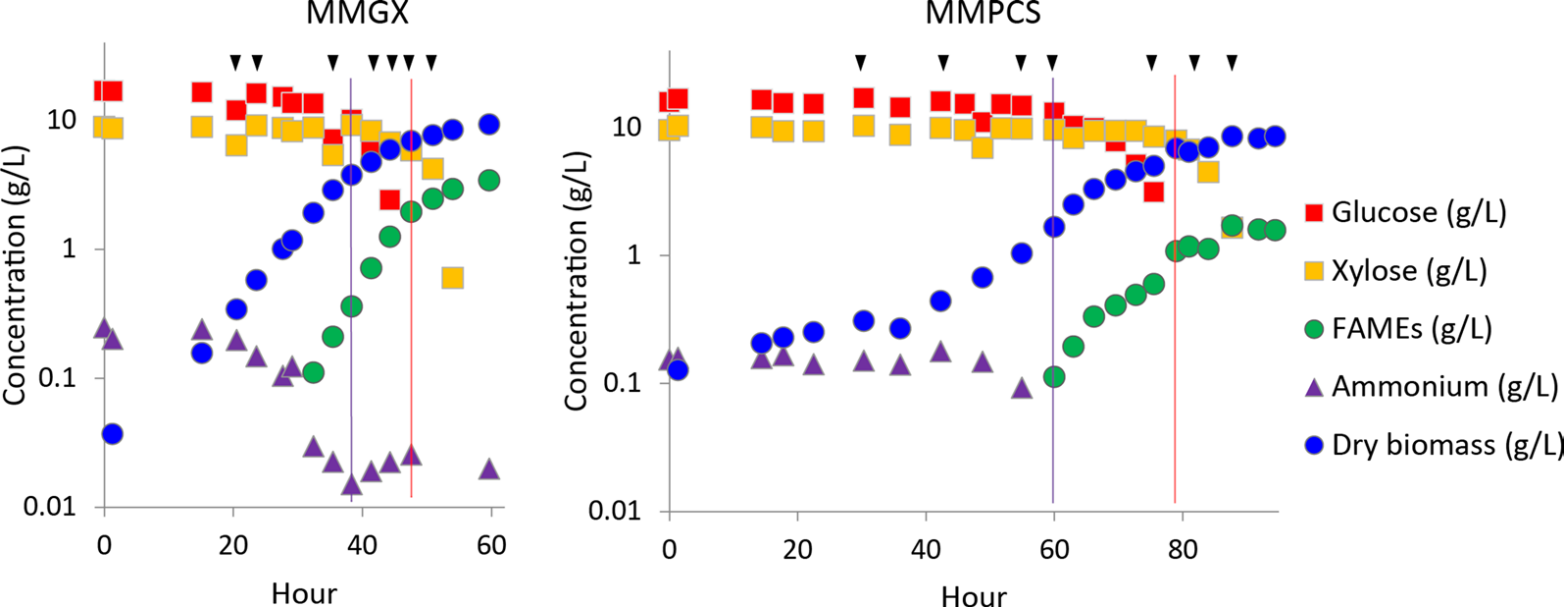
Physiological analysis of batch cultivation in a 20-L bioreactor. *L. starkeyi* grown with equivalent amounts of the sugars, glucose and xylose, supplied in either purified form (MMGX) or from PCS (MMPCS) was cultivated in nitrogen-limiting conditions and sampled at physiologically equivalent stages of growth and lipid accumulation. Triangles indicate time-points assessed by transcriptomics. Purple and red lines indicate the point at which glucose and ammonium reach undetectable levels

We designed a batch bioreactor cultivation strategy to assess the effect of inhibitory compounds present in PCS on nitrogen limitation-induced lipid production by the oleaginous yeast *L. starkeyi*. We utilized a minimal medium (MM) formulated with nitrogen (ammonium chloride) as the growth-limiting nutrient (Fig. 2) and cultivated the yeast in a 20-L bioreactor with either PCS (MMPCS) or an equivalent amount of glucose and xylose (MMGX) as the source of carbon. Online physiological monitoring indicates slower growth of *L. starkeyi* with PCS as the carbon source than with purified sugars (Fig. 3). To account for this, we collected samples for in-depth analysis at equivalent physiological time-points post-inoculation. These included sampling during biomass production and around the time of nitrogen depletion which coincides with the onset of rapid lipid accumulation (Fig. 3).

### Metabolic transitions during batch lipid accumulation

**Fig. 4:**
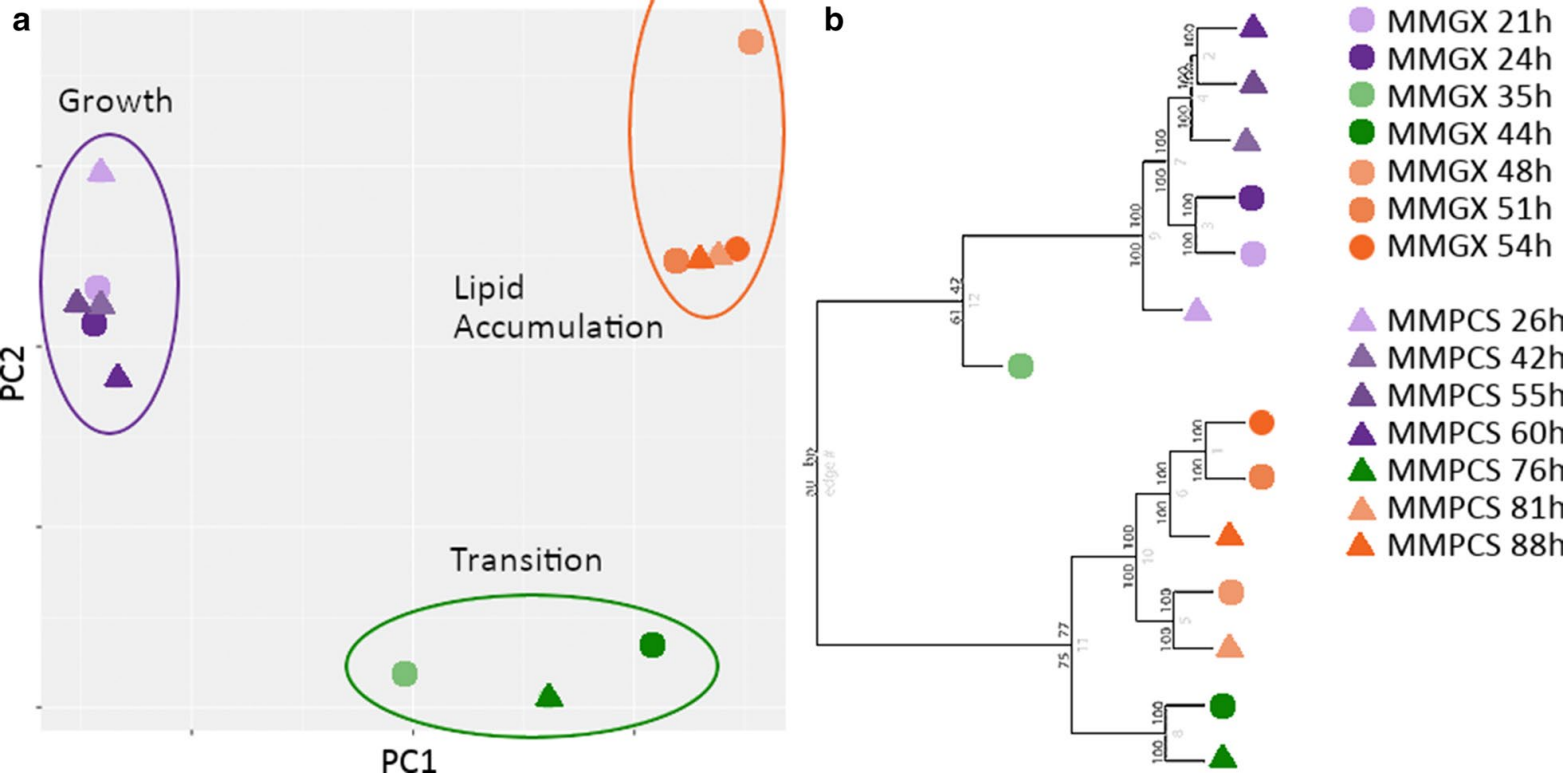
Clustering of global transcriptome profiles during batch cultivation. **a** Principle Component Analysis using singular value decomposition. **b** Hierarchical clustering using the average method of correlation distances. Bootstrap values were calculated from 1000 replicates. The samples can be distinguished into three phases; growth, transition, and lipid accumulation based on similarity of the transcriptome profiles

Gene expression in *L. starkeyi* during batch bioreactor cultivation was quantified by RNA-seq (Additional file 1). Global gene expression profiles for *L. starkeyi* grown in both MMPCS and MMGX were hierarchically clustered. The gene expression profiles clustered into three groups that correspond to physiological phases of the batch culture (Fig. 4). These include growth phase and initial biomass accumulation, a transition phase, and then accumulation of lipids. The early growth phase and late lipid accumulation phase expression profiles cluster tightly, while the transition phase profiles cluster more poorly. This indicates a relatively constant pattern of expression during the growth and lipid accumulation phases with a highly dynamic transition in-between.

**Fig. 5:**
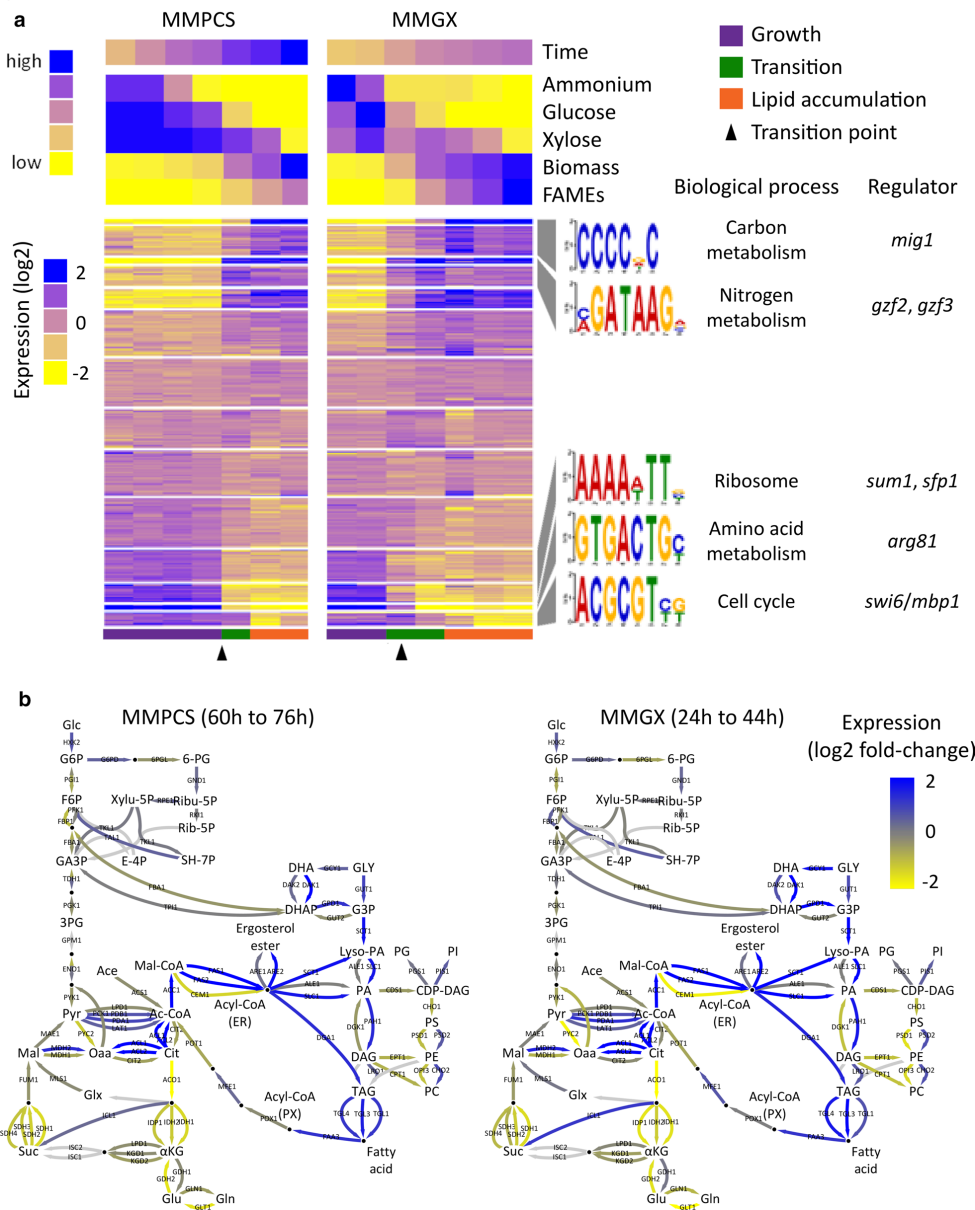
Transcriptome remodeling during the transition to lipid accumulation. **a** Transcriptome profiles from *L. starkeyi* growing on either MMPCS or MMGX were k-means clustered and ordered based on average slope of the fold-change. Specific DNA motifs are enriched in the promoters of genes in clusters with the most positive and negative fold-change slopes. Enriched biological process gene ontology terms were identified from groups of genes with the specific DNA motifs in their promoters. The specific DNA motifs are associated with transcriptional regulators in *S. cerevisiae*. **b** Expression fold-change values from the transition point mapped to central metabolism pathways and lipid biosynthesis pathways are highly similar for *L. starkeyi* grown on MMPCS and MMGX

In both the MMPCS and MMGX medium, the transition phase is associated with depletion of ammonium and glucose and results in similar patterns of gene expression change (Fig. 5). Gene ontology (GO) term enrichment analysis was performed to identify specific processes, functions, and compartments associated with the up- and down-regulated clusters of genes (Table 2). Genes involved in transport and carbohydrate metabolism are up-regulated during the transition suggesting that the cells are beginning to scavenge nutrients and engage in a broader range of metabolic activities. Ribosomal and translation associated genes are particularly down-regulated at the transition to lipid accumulation as would be expected when nitrogen for de novo amino acid biosynthesis is depleted.

**Table 2:** Gene ontology term enrichment during transition to lipid accumulation

GO term	Fold enrichment	Corrected *p* value
*Up-regulated gene clusters*		
Integral to membrane	1.98	7.81E−27
Transporter activity	2.40	3.14E−23
Oxidoreductase activity	1.88	1.13E−12
l-arabinose isomerase activity	2.86	7.23E−12
Carbohydrate transport	2.53	8.91E−09
Sugar:hydrogen symporter activity	2.65	1.30E−08
Carbohydrate metabolic process	2.23	2.81E−07
Nucleobase, nucleoside, nucleotide and nucleic acid transport	3.71	1.25E−04
Transcription factor activity	1.75	7.26E−04
Hydrolase activity, hydrolyzing *O*-glycosyl compounds	2.45	7.01E−03
*Down-regulated gene clusters*		
Structural constituent of ribosome	4.35	1.57E−57
Translation	3.59	1.13E−56
RNA binding	2.97	1.41E−10
Protein folding	2.99	1.05E−06
Unfolded protein binding	3.55	2.65E−06
rRNA processing	4.11	3.51E−04
Ribosome biogenesis and assembly	4.79	7.78E−04
ATP binding	1.41	2.64E−03
Translational elongation	4.32	5.72E−03
Aminoacyl-tRNA ligase activity	2.51	3.48E−02

Combined analysis of the clusters of genes up- and down-regulated during the transition. *p* value was corrected for multiple comparisons using the Bonferroni method

The initiation of lipid accumulation involves dynamic changes in expression of metabolic genes that are consistent, regardless of growth on PCS or purified glucose and xylose (Fig. 5). Genes necessary for assimilation of ammonium (*glt1* and *gdh2*) are down-regulated as are the genes directing pyruvate toward alpha-ketoglutarate (*pyc2*, *cit2*, *aco1*, *idp1*, *idh1*, *idh2*) through the TCA cycle. Genes for the production of fatty acids from citrate are up-regulated (*acl1*, *acl2*, *acc1*, *fas1*, *fas2*) as are the genes for de novo synthesis of triglycerides via incorporation of fatty acids onto a glycerol-3P backbone (*sct1*, *slc1*, *pah1*, *dga1*). Interestingly, genes that breakdown triglycerides are also up-regulated (*tgl1*, *tgl3*, *tgl4*, *faa3*) as was found for nitrogen limitation in *Y. lipolytica* [38]. The conserved phenomenon of simultaneous triglyceride production and utilization remains perplexing. However, deletion of *tgl4, pex10,* or *mfe1* did not increase lipid accumulation in *L. starkeyi* (Dai et al.; unpublished data) suggesting that breakdown of lipids via beta-oxidation is insignificant during nutrient limitation, consistent with early findings on lipid turnover during carbon limitation [39].

### Regulation of the transition to lipid accumulation

The ammonium depletion induced transition to lipid accumulation is associated with global transcriptome remodeling. To dissect this further, genes were divided into k-means clusters based on their expression pattern across both medium types (Fig. 5). The promoter regions of the genes within each cluster were analyzed for over-represented DNA sequence motifs to discover specific transcriptional regulators associated with the transition. In general, genes that are up-regulated during the transition to lipid accumulation are enriched for 5′-CCCCDC-3′ (*p* < 1E−27) and/or 5′-GATAAG-3′ (*p* < 1E−9) motifs in their promoters. In both the MMPCS and the MMGX medium nitrogen is depleted and is associated with up-regulation of genes with 5′-GATAAG-3′ motifs in their promoters followed by depletion of glucose and up-regulation of genes with 5′-CCCCDC-3′ motifs in their promoters (Fig. 5a).

**Fig. 6:**
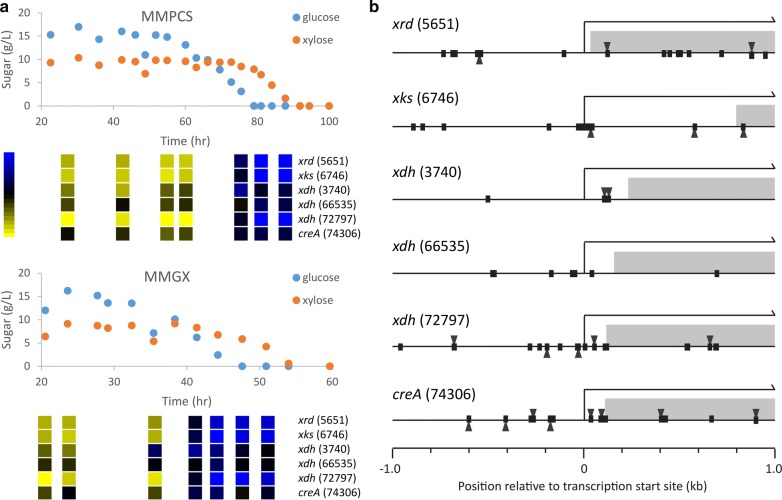
Regulation of the xylose utilization pathway. **a** Comparison of xylose utilization gene expression and extracellular sugar concentration during batch bioreactor growth. *xrd*, xylose reductase; *xdh*, xylitol dehydrogenase; *xks*, xylulose kinase; *creA*, carbon response element binding transcription factor. JGI protein IDs are given in parentheses. **b** Predicted binding sites for carbon response binding transcription factors (5′-CCCC-3′; rectangles) and the extended motif identified as enriched in the promoters of upregulated genes (5′-CCCCDC-3′ triangle) around the transcription start site of xylose utilization genes. Coding regions are indicated by gray bars

The 5′-CCCCDC-3′ motif is associated with genes involved in carbon metabolism and is similar to the sequence bound by Mig1p in *S. cerevisiae* [40] and CreAp in filamentous fungi such as *Aspergillus nidulans* [41]. This motif and its bound transcriptional regulators are involved in the utilization of alternative carbon sources and silencing of genes via carbon catabolite repression across fungal species [42–46]. In both medium types, xylose utilization begins just prior to, or concurrent with, depletion of glucose (Figs. 3, 6) when genes involved in the xylose utilization pathway (xylose reductase, xylulose kinase, and xylitol dehydrogenase) are up-regulated (Fig. 6) during the major transcriptional shift that occurs when lipids begin to accumulate. Analysis of the promotor regions revealed 5′-CCCCDC-3′ motifs in close proximity to the predicted transcription start site in the most dynamic xylose utilization genes (*xrd*, 5651; *xks*, 6746; *xdh*, 3740; *xdh*, 72797), while *xdh* (66535) does not change much in expression at this transition and does not exhibit the 5′-CCCCDC-3′ motif in its promoter (Fig. 6). This suggests that carbon catabolite repression regulates utilization of xylose in *L. starkeyi*. Expression of the *creA* homolog (74306), while not as dynamic as the xylose utilization genes, increases significantly at the transition point suggesting that it is self-regulated as would be expected with the large number of 5′-CCCCDC-3′ sites in close proximity to its transcription start site (Fig. 6). Increased expression of the repressor responsible for carbon catabolite repression at the point when repression is lifted is somewhat counterintuitive but may allow for abundant *creA*-derived transcript or protein to respond rapidly to the presence of a preferred carbon source through translational or post-translational mechanisms. In *Aspergillus nidulans*, import of CreAp into the nucleus is dependent on phosphorylation by Protein Kinase A [47]; while in *S. cerevisiae,* Mig1p is phospho-regulated by the Snf1p kinase [48] suggesting that transcriptional regulation of *creA* may play only a minor role.

The 5′-GATAAG-3′ motif is similar to that bound by the GATA binding family of zinc-finger transcription factors. In *S. cerevisiae,* this includes four transcription factors (*gat1* and *gln3*; *dal80* and *gzf3*) that activate and repress nitrogen assimilation genes, respectively [42, 49–52]; while the oleaginous yeast *Y. lipolytica* has two GATA binding zinc-finger transcription factors that activate (*gzf2*) and repress (*gzf3*) nitrogen metabolism genes [44] in response to alternative nitrogen sources, similar to the case in filamentous ascomycetes, such as *A. nidulans* and *Neurospora crassa* [53–55]. The *L. starkeyi* genome encodes one gene each that belongs to the nitrogen responsive activator (Lipst1_1 protein ID 424) and repressor (Lipst1_1 protein ID 45435) families [44] as well as homologs of iron responsive GATA binding transcription factors [56] (Lipst1_1 protein ID 73379) and the light-responsive GATA binding transcription factor *wc*-*2* [57] (Lipst1_1 protein ID 71310). None of these transcription factors are differentially expressed during the transition to lipogenesis.

Genes that are down-regulated after the transition phase have 5′-AAAAWTT-3′ (*p* < 1E−35), 5′-GTGACTG-3′ (*p* < 1E−8), and 5′-ACGCGT-3′ (*p* < 1E−5) motifs overrepresented in their promoter regions. Gene ontology term analysis of genes with these DNA sequence motifs in their promoters found that they are associated with the ribosome, amino acid metabolism, and the cell cycle, respectively. All three of these motifs bear a similarity to binding sites of transcriptional regulators in *S. cerevisiae*. These include Sum1p [58, 59], Sfp1p [60–62], the amino acid metabolism regulator Arg81p [63, 64], and the Swi6p/Mbp1p cell cycle regulatory complex [65, 66] (Fig. 5). This suite of motifs enriched in down-regulated genes emphasizes the interplay between depletion of nitrogen, translation, and cell cycle regulation, and suggests that *L. starkeyi* responds to nitrogen depletion by reducing amino acid anabolism and translation of new proteins, as well as cell division by halting at the G1/S check point. Analysis of the response to nitrogen depletion in the oleaginous yeast *Y. lipolytica* found a similar response [38, 67] that is dependent on the G1 cyclin *cln3* in *S. cerevisiae* [68].

### Differential analysis of clean sugars and PCS

Genes significantly altered in expression between the MMPCS and MMGX medium (adjusted *p* value < 0.01 and fold-change > 2) were identified for each of the three phases of batch cultivation (growth, transition, and lipid accumulation) to assess the effect of additional compounds present in the PCS. During growth phase, 73 genes were identified as differentially expressed; while fewer were found during the transition (16) and lipid accumulation (28) phases suggesting that the early stages of growth are when the majority of processes specific to the MMPCS medium occur. Three genes are down-regulated and 55 up-regulated specifically during the growth phase in MMPCS. All three down-regulated genes are transporters; while the up-regulated genes are generally uncharacterized but enriched for proteins involved in regulation of oxidoreductase activity (Lipst1_1 protein ID 68636, 76455, 155344, 243010, 2656, 97553, 76225, 58746, 163820, 5102, 78080, 146211, 2092, 146211, 76363, 117871, and 323398) and transport (3675, 7303, 114515, 76372, 52551, and 7145).

**Fig. 7:**
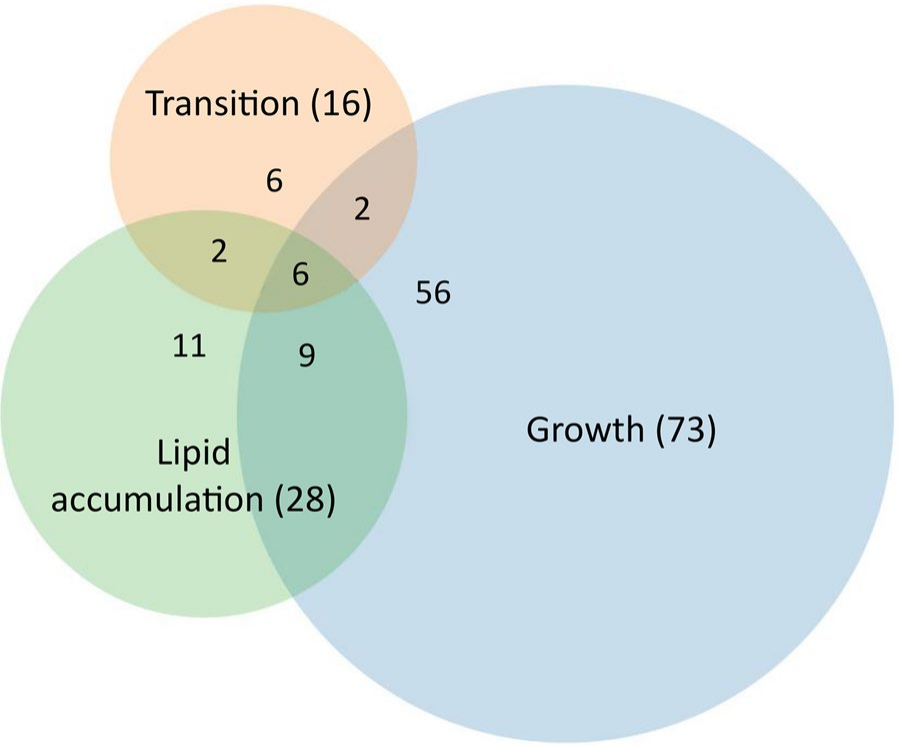
Genes differentially expressed in MMPCS versus MMGX. Genes altered significantly in expression during each phase of the batch bioreactor cultivation were identified with an adjusted *p* value < 0.01 and fold-change greater than two. Numbers in parentheses represent the total number of differentially expressed genes during each phase

Most of the differentially expressed genes in the transition and lipid accumulation phases are found in more than one phase (Fig. 7). These 19 genes that are differentially expressed in more than one of the phases are indicative of a longer-term PCS-specific response. Examination of this set of genes revealed that pyruvate decarboxylase (Lipst1_1 protein ID 70370) is down-regulated in MMPCS (Table 3); while many more genes are up-regulated, including a variety of enzymes and transporters. In *S. cerevisiae, pdc1* is important for fermentation to ethanol in anaerobic conditions as well as resistance to a variety of chemicals [69]. We have observed ethanol production by *L. starkeyi* in oxygen-limited conditions (0.18 ± 0.03 wt% after 5 days in shake flasks) but not in aerobic conditions. However, further work will need to be done to understand if and why *L. starkeyi* may be undergoing a fermentation response in MMGX.

**Table 3:** Genes differentially expressed in MMPCS versus MMGX

GeneID	Description	Growth	Fold-change (log2)	
*L. starkeyi*			Transition	Lipid accumulation
201508	Conserved hypothetical	*8.51*	*4.64*	*2.76*
5082	Cinnamyl alcohol dehydrogenase	*5.64*	*4.81*	*4.16*
201186	Orphan	*6.79*	3.54	*3.49*
170668	Orphan	*5.59*	*3.55*	*3.20*
6033	Polyamine transporter	*4.48*	*3.89*	*2.95*
68709	Aldehyde dehydrogenase (toxD)	*4.87*	*3.69*	2.39
3194	Nucleoside diphosphate sugar epimerase	*5.52*	2.48	*2.07*
73608	Nitroreductase	*2.12*	*4.17*	*3.19*
291194	ABC transporter	*3.56*	*2.97*	2.69
73626	Conserved hypothetical	*4.94*	1.83	*1.99*
3195	Oxidoreductase	*4.50*	1.79	*2.20*
74501	Conserved hypothetical	*2.41*	3.32	*2.68*
258339	MFS transporter	*2.62*	*3.23*	*2.49*
76819	NAD(P)H-dependent FMN reductase	*4.15*	2.94	*0.73*
5601	Monocarboxylate transporter	*2.90*	1.90	*1.65*
119382	Conserved hypothetical	*3.60*	0.80	*1.69*
68269	NADH dehydrogenase	0.21	*2.77*	*1.83*
108039	Orphan	**−** ***2.56***	− 1.79	**−** ***1.43***
70370	Pyruvate decarboxylase	− 0.73	**−** ***2.30***	**−** ***1.10***

Genes differentially expressed during more than one phase of growth, transition, or lipid accumulation. Significantly up-regulated genes are italics, while down-regulated genes are bold italics

The persistently up-regulated genes include an aldehyde (Lipst1_1 protein ID 68709) and alcohol dehydrogenase (5082) with homologs known to function in detoxification of cinnamic acids, furfural, and hydroxymethylfurfural [70–72]. Four transporters are up-regulated including two with unknown functions that are homologous to a multi-drug resistance transporter (291194), and a low-affinity ammonium transporter (258339) [73]. The polyamine transporter (6033) similar to *tpo1* [74] is up-regulated as is a predicted monocarboxylate transporter (5601). However, the homolog of 5601 in *S. cerevisiae*, *mch5*, is involved in riboflavin rather than monocarboxylate transport [75, 76] suggesting a role for riboflavin or FAD-dependent processes in general in utilization of MMPCS medium. Intriguingly, genes with a predicted nucleoside diphosphate sugar epimerase (3194) and an oxidoreductase (3195) domain are both up-regulated, particularly during early growth phase and are next to each other on the chromosome though expressed from different promoters. A handful of other genes with poor functional characterization are strongly up-regulated in MMPCS including a predicted nitroreductase, an NAD(P)H-dependent FMN reductase, an NADH dehydrogenase, and four conserved genes of unknown function (Table 3).

Three genes are down-regulated and three up-regulated specifically during the transition phase in MMPCS. Two metal transporters (Lipst1_1 protein ID 71180 and 89156) with homology to the high-affinity zinc transporter *zrt1* [77] are more highly expressed in the MMGX medium suggesting that PCS has a higher concentration of zinc, which regulates expression of this transporter in *S. cerevisiae* [78]. Genes specifically up-regulated during the transition phase include a predicted galactokinase (75890) and l-arabinose isomerase (89272). These genes are involved in utilization of the sugars galactose and arabinose that are likely to be present in small quantities in MMPCS but not MMGX.

Four genes are down-regulated and seven up-regulated specifically during the lipid accumulation phase in MMPCS. A predicted pantothenate MFS transporter (Lipst1_1 protein ID 716) is down-regulated, again suggesting that MMPCS contains a higher concentration of a trace nutrient, in this case pantothenate. A predicted cyclopropane-fatty-acyl-phospholipid synthase (4209) is down-regulated. In *Escherichia coli,* this lipid modification is important for acid and freeze–thaw tolerance suggesting that plasma membrane remodeling may be important for growth on PCS [79, 80]. Genes specifically up-regulated during the lipid accumulation phase include an uncharacterized cytochrome p450 (115210) and a phenylacrylic acid decarboxylase (76260), the homolog (*pad1*) of which confers resistance to cinnamic acids in *S. cerevisiae* [81]. A predicted dienelactone hydrolase orthologous to *aim2*, which is involved in mitochondrial function but does not have a defined role [82], is also up-regulated. In bacteria, genes with this domain play a role in detoxification of chloroaromatics [83, 84] suggesting another class of molecules that could play a role in inhibition of growth on PCS.

## Discussion

Robust growth of microorganisms and carbon-efficient production of biofuels using lignocellulosic biomass as a substrate are a challenge to the development of an economically viable and sustainable bio-economy. Efficient bioconversion processes require physical pretreatment and enzymatic hydrolysis of lignocellulosic biomass to release simple sugars for rapid conversion. These sugar-rich hydrolysates generally contain plant-derived breakdown products that inhibit the growth of microorganisms and overall bioconversion efficiency. We found that acetic acid and furfural present at levels found in PCS inhibit the growth of the robust lipid producer *L. starkeyi* when challenged to grow from a low initial cell density in minimal medium (Fig. 1). Growth inhibition by these compounds in PCS during batch bioreactor cultivation is overcome, however, possibly due to inoculation at a higher initial cell density (Fig. 3).

Growth on PCS presents a challenge to most microorganisms due to various toxic compounds. *L. starkeyi* is able to overcome this challenge, grow robustly, and accumulate lipids in a timely manner on PCS (Fig. 3). To further our understanding of the impact of inhibitory compounds in PCS, we compared *L. starkeyi* grown on PCS versus a clean glucose and xylose mixture. We analyzed expression of RNA during batch cultivation to identify genes associated with growth in the presence of inhibitors in PCS, so that a more robust strain can be engineered. Resistance could manifest itself as the ability to truly catabolize inhibitors such as acetate and phenolics from lignin breakdown, or inactivate the inhibitory functional groups by reduction of harmful aldehyde and ketone groups (furfural, HMF, acetaldehyde) to alcohols) that can cause cross-linking and inactivation of biological macromolecules, especially proteins, or continuous export of inhibitors that cross the plasma membrane.

We found that while the amount of time required for growth and production of lipids was extended on PCS (Fig. 3), the overall trends in gene expression and their relation to nutrient depletion were highly similar to that of growth on clean sugars (Figs. 4, 5). We, thus, identified and compared equivalent physiological conditions during growth on PCS and clean sugars rather than equivalent points in time during the batch lipid accumulation runs. Clustering identified two robust states, one associated with early growth and another associated with lipid accumulation following depletion of ammonium and glucose. Understanding the transition to lipid accumulation is particularly interesting in oleaginous biofuel production hosts such as *L. starkeyi*. Early work on lipid accumulation identified limitation of macronutrients including nitrogen, phosphorus, and sulfur in the presence of excess carbon as a readily controllable method to induce lipid accumulation [23, 85–88]. Here, we identified promoter motifs and enriched GO terms associated with gene sets that tend to be up- and down-regulated between the growth and lipid accumulation phases. Up-regulated genes tend to be enriched for motifs in their promoters associated with carbon and nitrogen catabolite repression (Fig. 5). In *S. cerevisiae,* these processes are controlled by sequence specific transcription factors [42]. At the onset of lipid accumulation, genes silenced by carbon and nitrogen catabolite repression are activated suggesting derepression of these processes in an attempt by the cells to scavenge additional sources of carbon and nitrogen. Induction of the repressed genes occurs prior to depletion of glucose and is associated more with depletion of nitrogen suggesting nitrogen starvation as the initial motivator for the change in gene expression. Interestingly, genes typically silenced by carbon catabolite repression in ascomycetes, such as the genes for xylose utilization [89–92], are activated prior to depletion of glucose. Xylose utilization also begins prior to depletion of glucose. We examined these genes specifically and found that they are enriched for motifs near their transcription start site associated with carbon but not nitrogen catabolite repression (Fig. 6), further confirming that carbon catabolite repression is controlling their expression and is lifted prior to complete glucose depletion. The interplay between carbon and nitrogen catabolite repression has not been fully elucidated, particularly during dynamic processes. In the oleaginous yeast *Y. lipolytica,* genes with the carbon catabolite repression motif in their promoter regions are silenced when nitrogen catabolite repression is lifted [38] and this effect is reproduced by deletion of *gzf3*, which is required for silencing of genes via nitrogen catabolite repression [44]. This is opposed to the findings here, suggesting that the presence of xylose in these experiments is signaling the cells to activate genes silenced by carbon catabolite repression prior to complete utilization of glucose.

Analysis of differentially expressed genes found that most are identified specifically during growth phase. This observation supports the notion that PCS has a greater impact on the growth phase of the organism, which requires a large array of complex biosynthetic reactions, than on the lipid accumulation phase under nitrogen starvation, where cell growth has basically ceased (Fig. 7). The majority of the differentially expressed genes are up-regulated demonstrating that growth on MMPCS requires additional metabolic activities. During growth phase, we identified a variety of uncharacterized enzymes and conserved proteins of unknown function that may play a role in utilization and/or detoxification of chemical components of PCS. Three of the up-regulated enzymes are similar to those known to play a role in detoxification in other organisms (Lipst1_1 protein ID 68709, 5082, and 76260). These include two medium-chain dehydrogenase/reductase family members belonging to the cinnamyl alcohol dehydrogenase family (5082) and the quinone oxidoreductase family (68709), respectively. In *S. cerevisiae,* the cinnamyl alcohol dehydrogenases (*adh6* and *adh7*) exhibit broad substrate specificity, and are involved in lignin degradation and in detoxification of hydroxymethylfurfural [72, 93, 94]. The quinone oxidoreductase family protein is homologous to the broad substrate aldehyde dehydrogenase (*YNL134c*) in *S. cerevisiae*, which is up-regulated in the presence of furfural and detoxifies it to 2-furanmethanol [70]. It is likely that *L. starkeyi* detoxifies PCS using these enzymes.

## Conclusions

In summary, we compared the physiology and gene expression of *L. starkeyi* during batch cultivation on “clean” sugars and the more complex carbon source PCS. Overall growth and metabolism are similar on the two carbon sources with the primary difference occurring during early growth phase when genes specific to growth on PCS are up-regulated. Some of the genes identified are implicated in detoxification of compounds present in PCS in other fungi but most are uncharacterized and need to be tested specifically for function. This work provides a foundation for understanding the metabolic capabilities necessary for growth on a complex and potentially toxic substrate being considered for industrial use as a lignocellulosic biofuel feedstock.

## Additional file

**Additional file 1.** Excel file that contains metabolite quantification during the bioreactor runs as well as raw RPKM values from RNA-seq. Genes that are differentially expressed between the MMPCS and MMGX bioreactor runs during at least one phase are listed along with their fold-change values.

## Data Availability

All data generated or analyzed during this study are included in this published article and its supplementary information files, or in a public archive. Raw transcriptome data have been deposited at NCBI SRA under Bioproject PRJNA542162 (accession numbers SRR9036793, SRR9036792, SRR9036791, SRR9036790, SRR9036789, SRR9036788, SRR9036787, SRR9036786, SRR9036785, SRR9036784, SRR9036783, SRR9036782, SRR9036781, SRR9036780).

## Abbreviations

GC: gas chromatography
GO: gene ontology
GC: gas chromatography
GX: glucose and xylose
HMF: hydroxymethylfurfural
HPLC: high-pressure liquid chromatography
MM: minimal medium
PCS: pretreated corn stover
FAMEs: fatty acid methyl esters
